# Chromatin Structure Around Long Non-Coding RNA (lncRNA) Genes in *Schistosoma mansoni* Gonads

**DOI:** 10.3390/ncrna11020025

**Published:** 2025-03-12

**Authors:** Ronaldo C. Augusto, Thomas Quack, Christoph G. Grevelding, Christoph Grunau

**Affiliations:** 1 IHPE, Université de Perpignan Via Domitia, CNRS, IFREMER, Université de Montpellier, 66860 Perpignan, France; ronaldo.augusto@univ-perp.fr; 2 Institute for Parasitology, BFS, Justus Liebig University, 35392 Giessen, Germanychristoph.grevelding@vetmed.uni-giessen.de (C.G.G.)

**Keywords:** *Schistosoma mansoni*, posttranslational histone modifications, long non-coding RNA, chromatin, gonads

## Abstract

In this study, we employed a total of eight distinct modifications of histone proteins (H3K23ac, H3K27me3, H3K36me3, H3K4me3, H3K9ac, H3K9me3, H4K12ac, and H4K20me1) to discern the various chromatin colors encompassing lncRNA genes in both mature and immature gonads of the human parasite *Schistosoma mansoni*. Our investigation revealed that these chromatin colors exhibit a tendency to aggregate based on the similarities in their metagene shapes, leading to the formation of less than six distinct clusters. Moreover, these clusters can be further grouped according to their resemblances by shape, which are co-linear with specific regions of the genes, and potentially associated with transcriptional stages.

## 1. Introduction

Schistosomiasis, a parasitic disease caused by members of the *Schistosoma* genus, represents a significant public health burden in tropical and subtropical regions, affecting 250 million people worldwide. The life cycle of these parasitic flatworms involves complex interactions with both intermediate and definitive hosts, resulting in chronic infections that can lead to severe morbidity and mortality if left untreated. Despite its substantial impact on global health, schistosomiasis has received relatively limited attention in comparison to other neglected tropical diseases, and the intricacies of its molecular mechanisms remain incompletely understood [1].

Schistosomes are exceptional among platyhelminths, as they are the only that have evolved separate sexes. Additionally, their sexual biology is nearly unique, as the maturation of the female gonad requires continuous pairing contact with a male partner [2,3,4,5]. Parasite pairing induces the final differentiation of the ovary, which, in unpaired females consists of stem cell-like, undifferentiated oogonia that do not enter meiosis. After pairing, however, mitoses of gonadal stem cells are induced, and part of the daughter cells start to differentiate entering meiosis [6,7]. These, and other changes, are accompanied by a remarkable growth of the paired female, and they are mediated by complex signaling systems as well as many genes expressed in a pairing-dependent manner, including gonadal genes. Although no morphological changes are observed following pairing, previous transcriptomics data of protein-coding genes of whole worms (and their gonads) revealed the influence of the pairing status on gene expression in males, including their testes [8,9].

Recent advances in genomics and transcriptomics have shed light on the role of long non-coding RNAs (lncRNAs) in the regulation of gene expression and the modulation of biological processes across various organisms. LncRNAs, a class of non-protein-coding transcripts with a length exceeding 200 nucleotides, have emerged as crucial players in diverse cellular and physiological functions, including development, differentiation, and disease pathogenesis [10,11,12]. Although the role of lncRNAs has been extensively investigated in various organisms, their involvement in schistosome biology remains largely unexplored territory.

Understanding the regulatory mechanisms governing schistosome lncRNA gene expression would allow for elucidating key aspects of their biology, including host–parasite interactions, immune evasion, and developmental transitions. In this context, the exploration of lncRNAs in schistosomes presents a promising avenue for uncovering novel regulatory elements and mechanisms that may contribute to the parasites’ adaptation and survival within their hosts.

In *S. mansoni*, lncRNAs have been shown to affect pairing status, survival, and reproduction in adult worms. They are also implicated in the formation of liver granulomas and the polarization of macrophages, providing a potential target for treatment [13,14]. Additionally, lncRNAs have been identified in different developmental stages of *S. mansoni*, suggesting their involvement in stage-specific gene expression and their potential as biomarkers [15,16]. Furthermore, the expression patterns of lncRNAs in *S. mansoni* have been characterized at the single-cell level, revealing tissue-specific expression and regulated expression programs [17].

Given the stage- and cell-specific expression of lncRNA and its sensitivity to an epigenetic stressor [18], we hypothesized that lncRNA gene function could be influenced by the chromatin structure around their genes. This study aimed to characterize these chromatin modifications around lncRNA genes and investigate their regulatory roles in *S. mansoni* gonads. Chromatin, the complex of DNA and proteins that constitutes the structural framework of eukaryotic genomes, plays a pivotal role in controlling gene function and maintaining genome integrity. Over the past few decades, extensive research has revealed the remarkable diversity and complexity of chromatin organization, leading to the emergence of the concept of “chromatin colors” or “chromatin flavors”. Through an analogy of the additive primary colors that can produce the full spectrum of visible colors, these metaphoric terms describe the idea that individual compositions of the chromatin can be combined into different functional states (“colors”) and can be adopted to orchestrate gene regulation and cellular identity [19].

Here, we used eight different posttranslational histone modifications (H3K23ac, H3K27me3, H3K36me3, H3K4me3, H3K9ac, H3K9me3, H4K12ac, and H4K20me1) to identify the chromatin colors surrounding lncRNA genes in gonads, ovaries, and testes, isolated from paired and unpaired *S. mansoni*. We found that chromatin colors cluster by similarity of their metagene shapes into less than six clusters, which can be grouped into similar shapes that appear to be associated with parts of the genes, the pairing status of the adults from which the gonads originated, and potentially with transcription steps.

## 2. Results

### 2.1. 500 bp Is the Optimal Bin Size for ChromstaR Analysis

ChromstaR [20] is an R-package for the analysis of ChIP-seq data based on the Hidden Markov Model (HMM). Regions of the genome that are enriched in specific histone modifications (“peaks”) are identified based on transition probabilities. ChromstaR allows us to combine individual histone modifications into combinational states (“chromatin colors”). It requires us to empirically establish a bin size prior to the full analysis. For this, we used comparison with other peakcalling methods and visual inspection under IGV for the relatively sharp H3K4me3 peaks. The best concordance of peak detection between MACS2, Peakranger, and ChromstaR was obtained at a bin size of 500 bp with the input BAM as reference. The visual inspection of peakcalling using MACS bedgraph, Peakranger, ChromstaR wig files, and BED files from ChromstaR revealed excellent concordance between peak representation and peak detection with these parameters. The metagene profiles were similar for the three methods and showed high similarity between replicates. The modified lines in the univariate fits of ChromstaR were flat, an empirical indicator for good model fitting. In conclusion, further analyses were performed with a binsize of 500 bp and input BAM as references.

### 2.2. There Are Sex-Specific Differences in Chromatin Profiles Around lncRNA Genes in Gonads of S. mansoni

Based on a previously established organ isolation method [21], we used pairing-experienced females (bisex females, bF) and males (bM), as well as used pairing-unexperienced females (sF, single sex female) and males (sM) for isolation of their gonads, ovaries from bF (bO) and sF (sO), and testes of bM (bT) and sM (sT). Next, we performed ChIP-seq with the abovementioned eight antibodies. We aligned the ChIP-seq reads to the genome and generated metagene profiles, i.e., log(observed/expected) of color enrichment spanning 2 kb upstream of the transcription start site (TSS) to 2 kb downstream of the transcription end site (TES). We noticed that some profiles had similar shapes and used unsupervised clustering to sort similar profiles into clusters. We allowed up to six clusters but only considered those with at least four colors. Cluster 0 contains the “flatliners”, i.e., no enrichment around lncRNA genes. The numbers of profiles in clusters are listed in Table 1.

**Figure 1:**
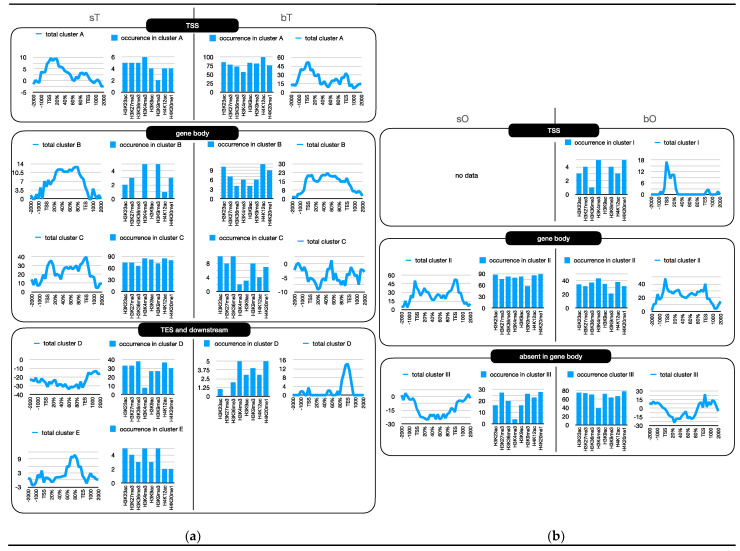
(**a**) Metagene profiles of testes of single-sex, pairing-unexperienced males (sT), and bisex, pairing-experienced males (bT), combined into clusters by shape and associated with gene parts. *X*-axis 2 kb upstream of TSS and 2 kb downstream of TES. *Y*-axis log(obs/exp) totaled over all profiles in the cluster. Histograms show the absolute number of occurrences of a histone modification in the chromatin colors. Squares surround groups of profiles with similar shapes. (**b**) Metagene profiles of ovaries of single sex, pairing-unexperienced females (sO), and bisex, pairing-experienced females (bO). *X*-axis 2 kb upstream of TSS and 2 kb downstream of TES. *Y*-axis log(obs/exp) totaled over all profiles in the cluster. Histograms show the absolute number of occurrences of a histone modification in the chromatin colors. Squares surround groups of profiles with similar shapes.

Through the visual inspection of cumulated log(obs/exp) metagene profiles, we realized that these profiles can be grouped by their shapes (Figure 1a,b). These groups were different in both sexes and varied between mature and immature gonads. We tentatively assigned the different cluster groups to parts of genes. It should be noted that clusters were relabeled during this annotation process, e.g., cluster A in bT has the same shape but not the same composition as cluster A in bT. We used letters and roman numerals in Table 2 and Figure 1 for better readability. The composition of the clusters and the correspondence between initial cluster numbers are presented in Table 1 and Table 2 and Figure 1, which can be found in Supplementary Figures S1 and S2. ChromstaR identified differential chromatin color peaks between gonads of pairing-experienced versus inexperienced worms: 94,148 regions in sF vs. bF, and 249,867 regions in sM vs. bM. This large number is to be expected since we used combinations of eight histone modifications.

Of the 16,583 lncRNA genes identified by Maciel et al. [22], 9489 (57%) overlapped by at least 1 bp with the differential chromatin regions in the ovaries, and 13,483 (81%) in the testes (Supplementary Files S2 and S3).

## 3. Discussion

Our results reveal a complex landscape of chromatin modifications surrounding long non-coding RNA (lncRNA) genes in the gonads of *S. mansoni*, with a theoretical capacity to generate 256 distinct “chromatin colors” through combinations of eight histone modifications. We used metagene projection, which is a computational technique used in epigenomics that simplifies the analysis of complex ChIP-seq data by reducing dimensionality and identifying overarching patterns across multiple genes. “Metagenes” are composite profiles summarizing histone modifications from groups of genes. These metagenes allowed us to project individual gene data onto a set of representative features, and to identify shared profiles by unsupervised clustering and overlay the resulting cluster with gene regions by visual inspection (Figure 1a,b).

Remarkably, in the gonads, we observed nearly the full spectrum of histone modification combinations around lncRNA genes, yet these many modifications cluster into fewer than six categories based on the similarities of their metagene profiles. This clustering seems to be associated with distinct steps of gene transcription, and represents a significantly reduced complexity compared to what might be expected. Such findings suggest a functional role for histone modifications in a regulatory mechanism that selectively employs certain histone modifications to orchestrate lncRNA transcription, e.g., we see in cluster B in the testes of virgin males (sT) that colocalizes with the gene body absence of H3K9ac and H3K36me3, and high occurrence of H3K4me3 and H3K9me3. In contrast, H4K12ac and H3K23ac are abundant in cluster B of testes from mating experienced males (bT). It also appears that there are two groups of genes: those with cluster B type combinations in the gene body, and others with the cluster C type. A limitation of the software we used is that we cannot associate the underlying groups of lncRNA genes with the clusters. Another peculiarity is observed within cluster D of bT and E of sT, in which there is an enrichment of chromatin colors that contain H3K4me3. H3K4me3 is typically found in the TSS of protein coding genes, suggesting a different function for lncRNA genes.

The discovery that the composition of chromatin colors varies between male and female gonads additionally indicates a sex-dependent difference. Furthermore, the pairing status of the worms adds another layer of complexity to the regulation of lncRNA transcription. The sexual dimorphism and pairing status specificity in chromatin modification patterns suggest that the regulation of lncRNA transcription is finely tuned to the physiological and developmental context of the parasite, especially with respect to the pairing status. The differential chromatin landscapes between males and females, and between different pairing statuses, imply that there is a strong epigenetic component in the form of histone modifications that might play a key role in mediating sex-specific and pairing-dependent gene expression programs. Differences between pairing–inexperienced and experienced worms, regarding the relative contributions of the eight histone modifications to the composition of the clusters, are particularly strong in the testes. This lends further support to the idea that inhibitors of histone modifying enzymes could be used for the pharmacological prevention of sexual maturation.

Our results suggest that the precise control of lncRNA transcription via specific patterns of histone modifications may represent a critical aspect of these regulatory functions. It is conceivable that aberrations in these chromatin modification patterns could lead to dysregulated lncRNA expression and contribute to developmental malfunction. Indeed, a recent functional study in *S. mansoni* identified lncRNA involvement in regulating cell proliferation in adults and gonads, as well as reproduction, including egg development [14]. Interestingly, recent publications have observed that some differentially expressed lncRNAs co-localize with protein-coding genes in the same cell clusters and co-expression modules, raising the hypothesis of the possible regulation of these genes by the lncRNAs [23]. Future studies modeling chromatin structures alongside the expression of lncRNAs and protein-coding genes are required in order to unravel the complex puzzle of gene expression in schistosomes.

In conclusion, our study provides additional compelling evidence for the functional role of histone modifications in the control of lncRNA transcription in *S. mansoni* gonads, revealing the possibility of a regulatory system that is sensitive to the parasites’ developmental and physiological context. These findings are starting points for new avenues of research that will lead to an understanding of the mechanisms of lncRNA gene regulation at the chromatin level, and their implications for development. They will also contribute to understanding the physiology of the parasite, and the control of schistosomiasis, a NTD for which new treatment concepts are urgently needed [24].

## 4. Materials and Methods

### 4.1. Life Cycle Maintenance of S. mansoni and Ethics Statement

To maintain the life cycle of the Liberian strain of *S. mansoni* [25], we used *Biomphalaria glabrata* as the intermediate host and hamsters as the final hosts. This is in accordance with the European Convention for the Protection of Vertebrate Animals Used for Experimental and Other Scientific Purposes (ETS No 123; revised Appendix A). The animal experiments were approved by the Regional Council Giessen (V54-19 c 20/15 c GI 18/10).

### 4.2. Organ Extraction and N-ChIP

To generate worm populations of one sex, we gave monomiracidial infections to snails, in which clonal cercarial populations (single-sex cercariae) develop, and which we subsequently used for final host infections to obtain pairing-unexperienced females or males. Mixed-sex worm populations were obtained by polymiracidial snail infections.

Gonad isolation was performed by a detergent- and enzyme-based isolation procedure [21] using about 50 pairing-experienced females and males, and 100 pairing-unexperienced females. These are much smaller than paired females, as are their ovaries. For the vitality check, 5–10 organs of each sample were stained with 10 µL Trypan Blue (0.4%; Sigma, St. Louis, MO, USA) and checked by phase-contrast microscopy (Olympus IX 81 microscope, Tokyo, Japan).

We performed native chromatin immunoprecipitation followed by sequencing (ChIP-seq), as described for *S. mansoni* in [26]. For each histone mark, we had two biological replicates for testes and ovaries from immature (sM and sF) and mature (bM and bF) adult worms. Immunoprecipitation was performed using antibodies against H3K4me3, H3K27me3, H3K9me3, H3K9ac, H3K23ac, H4K20me1, H4K12ac, H3K36me3 (Table 3).

The datasets used during the present study (BioProject ID: PRJNA1173904) are available from the corresponding author upon reasonable request. To ensure reproducibility in chromatin immunoprecipitation (ChIP) experiments, antibody and titration were performed prior to ChIP. This step is essential to establish the optimal antibody concentration, as an excess of antibody ensures efficient immunoprecipitation without affecting subsequent reproducibility [26]. Here, 20–40 μg of chromatin was used, and tested antibodies were titrated in the range of 2–20 μg. The reaction was incubated overnight at 4 °C with slow rotation. Then, the percentage of input recovery through qPCR careful standardization and biological duplicates were tested to confirm antibody specificity and the optimal antibody concentration (Supplementary Figures S1 and S2).

For each sample, we used a control without antibody to assess the nonspecific background (bound fraction) and input (unbound fraction).

### 4.3. Sequencing and Quality Control

ChIP products were all sequenced as paired-end 75 bp reads on an Illumina HiSeq 2500 at Wellcome Trust Sanger Institute (UK). Data processing was performed on a local GALAXY instance [27]. Read quality was verified using FastQC (V.0.72+galaxy1), ensuring a minimum average read quality score of 30 over 95% of their length, with no further cleaning needed. Sequences were aligned to the *S. mansoni* reference genome v7 with Bowtie v2.1 using end-to-end, sensitive, and gbar 4 parameters. BAM files were sorted and filtered for unique matches with samtools v1.3.1, removing PCR duplicates. Wiggle files were visualized with IGV.

### 4.4. Alignment and ChromstaR Optimization

Uniquely aligned reads for N-ChIP with antiH3K4me3 on two biological replicates of pairing experienced ovaries (bF) and pairing unexperienced ovaries (sF) were downsampled to 2.5 Mio reads per sample. The resulting BAM files were used as the input for peakcalling in MACS2 (effective genome size 350 Mb, building a model with lower mfold bound 5, upper mfold bound 50, band width for picking regions to compute fragment size 300), peak detection based on qvalue, minimum FDR (q-value) cutoff for peak detection 0.05), and peakranger 1.17 (peak detection mode at low resolution, read extension length 300 bp), both with input BAM DU Sm-SsOv-INPUT. ChromstaR was evoked with the default parameters and 250, 500, 750, and 1000 bp bin size, with or without input.

### 4.5. Metagene Analysis of lncRNA Genes and Clustering by Color

**Figure 2:**
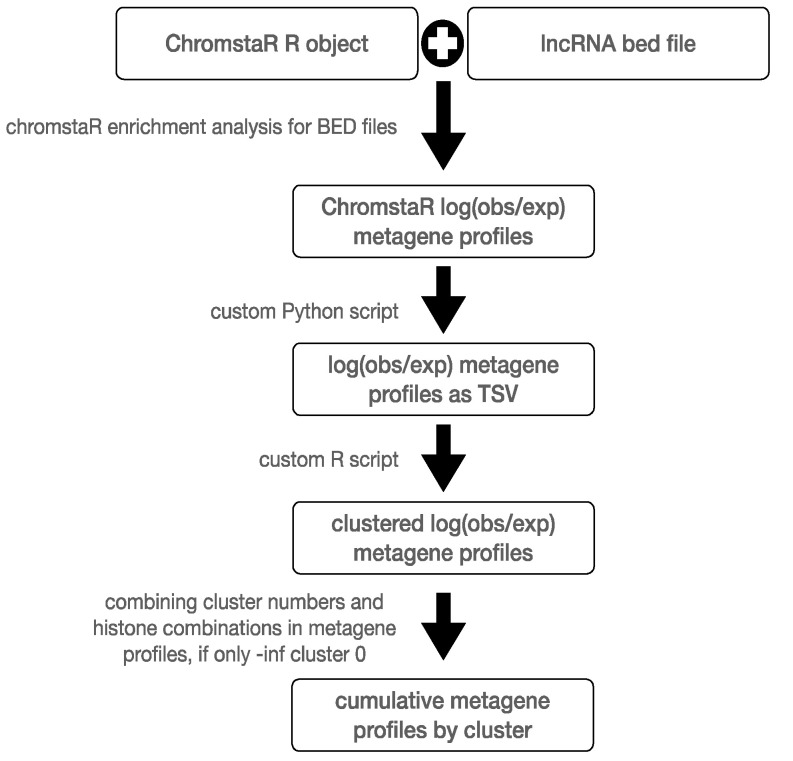
Schematic representation of the bioinformatics workflow used to perform clustering of metagene profiles. Custom Python (v.3.8.2) and R (v.R-4.2.3) scripts are provided as Supplementary Files S2 and S3.

Coding sequences of lncRNA were obtained as BED files from [12]. Metagene profiles were generated with the plotEnrichment function of ChromstaR using 8328 lncRNA coding genes on the plus strand. The metagene profiles of all combinations were exported from ChromstaR as individual TSV files. Hierarchical clustering was performed using the hclust function of R (v.R-4.4.3) (Figure 2).

## Figures and Tables

**Table 1:** Numbers of testes from single-sex males (sT; extracted from worms representing clonal, unisexual worm populations), bisex testes (bT; extracted from worms representing clonal, bisexual worm populations), single-sex ovaries (sO; extracted form ss females), and bi-sex ovaries (bO; extracted from bs females). The clusters that were used for further analyses are given in bold.

Cluster	sT	bT	sO	bO
0 (flatline)	19	58	45	37
1	**57**	**160**	**163**	**140**
2	**162**	**13**	**41**	**68**
3	**6**	**18**	2	**5**
4	2	1	1	1
5	**6**	**5**	1	1
6	4	1	1	2

**Table 2:** Assignment of clusters to part of genes (see also Figure 1). Abbreviations as in the main text.

Part of Gene	sT	bT	sO	bO
TSS	Cluster A	Cluster A		Cluster I
Gene body	Cluster B, C	Cluster B, C	Cluster II	Cluster II
TES and downstream	Cluster D, E	Cluster D		
Absent in gene body			Cluster III	Cluster III

**Table 3:** Details of the antibodies used for ChIP-seq of testes of single-sex pairing-unexperienced males (sT), and bisex pairing-experienced males (bT).

Antibody	Supplier	Catalog Number
H3K4me3	Diagenode	c15410003
H3K27me3	Diagenode	c15410069
H3K9me3	Abcam	ab8898
H3K9ac	Milipore	07-352
H4K20me1	Abcam	ab9051
H4K12ac	Abcam	ab46983
H3K36me3	Abcam	ab9050
H3K23ac	Milipore	07-355

## Data Availability

The datasets used during the present study (BioProject ID: PRJNA1173904) are available from the corresponding author upon reasonable request.

## Supplementary Materials

The following supporting information can be downloaded at: https://www.mdpi.com/article/10.3390/ncrna11020025/s1, Figure S1: Details of the antibodies used for ChIP-Seq of testes of single-sex, pairing-unexperienced males (sT), and bisex, pairing-experienced males (bT); Figure S2: Western blotting analysis of the antibodies used for ChIP-seq testes of *Schistosoma mansoni*; File S2: lncRNA in different regions in ovaries; File S3: lncRNA in different regions in testes.
