# Supplementary material for: Chromatin Structure Around Long Non-Coding RNA (lncRNA) Genes in Schistosoma mansoni Gonads
Source: Noncoding RNA. 2025 Mar 12;11(2):25. doi: 10.3390/ncrna11020025 (PMC11932260; doi:10.3390/ncrna11020025)
Supplement: Supplementary file 1 [file ncrna-11-00025-s001.zip › Supplementary Figures S1 and S2.pdf]

Supplementary Figure S1
Sample species: *S. mansoni*
ChIP method: N-ChIP

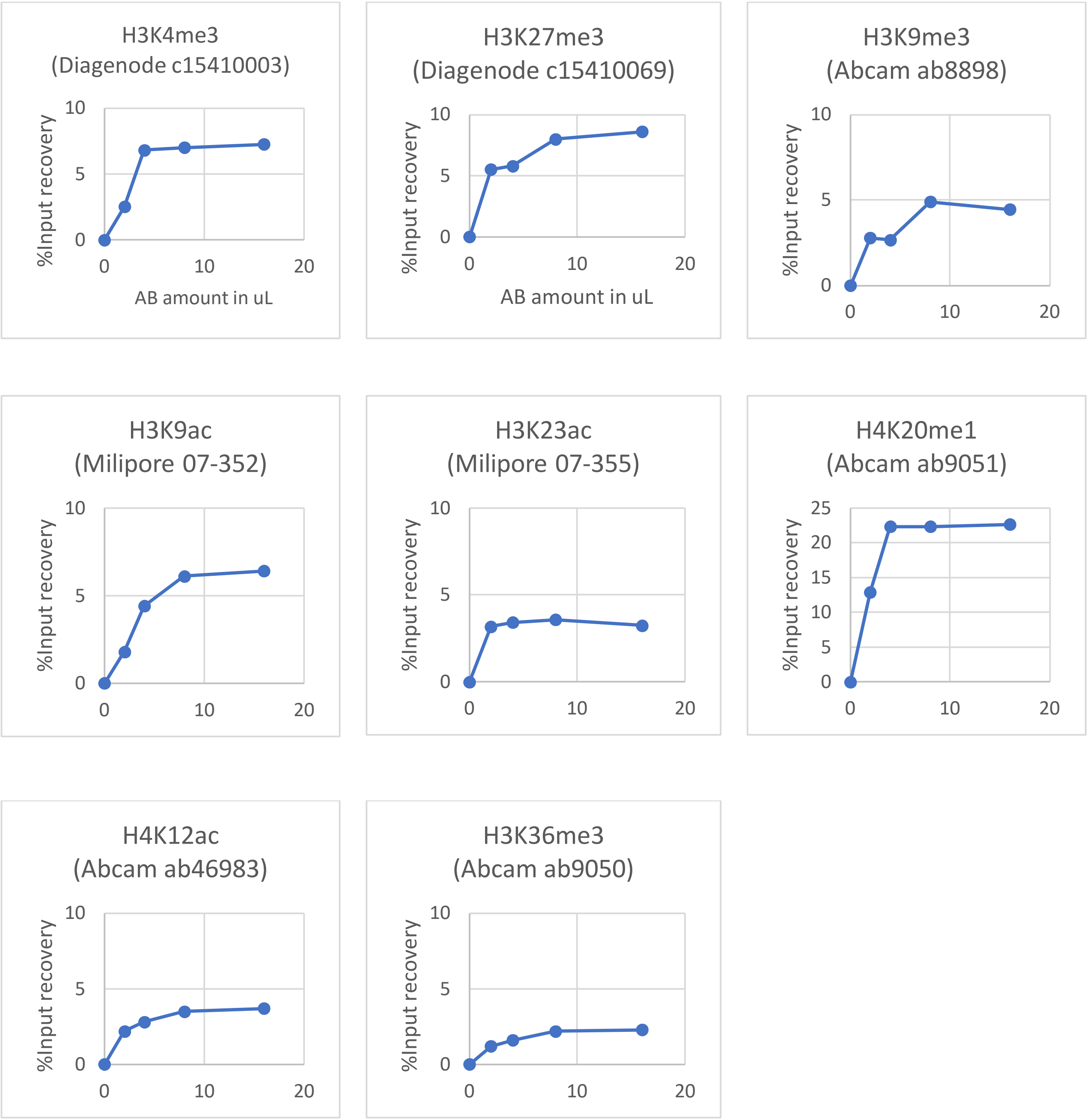

Supplementary Figure 1. Details of the antibodies used for ChIP-Seq of testes of single-sex, pairing-unexperienced males (sT), and bisex, pairing-experienced males (bT).

Supplementary Figure S2

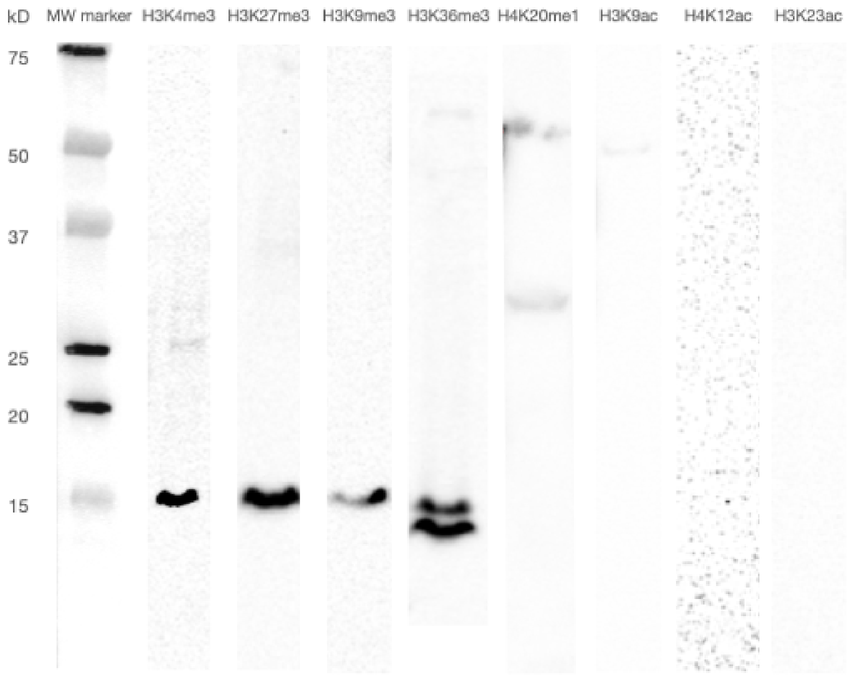

Supplementary Figure S2. Western blotting analysis of the antibodies used for ChIP-seq testes of *Schistosoma mansoni.*
